# Fertility-sparing surgery in advanced stage malignant ovarian germ cell tumor: a case report

**DOI:** 10.1186/s13256-017-1516-8

**Published:** 2017-12-17

**Authors:** Montassar Ghalleb, Hatem Bouzaiene, Skander Slim, Achraf Hadiji, Monia Hechiche, Jamel Ben Hassouna, Khaled Rahal

**Affiliations:** Surgical Oncology Department, Salah Azaiez Institute of Cancer, Tunis, Tunisia

**Keywords:** Fertility, Surgery, Malignant germ line tumor

## Abstract

**Background:**

Malignant ovarian germ cell tumor is a rare type of disease, which generally has a good prognosis due to the high chemosensitivity of this type of tumor.

Fertility preservation is an important issue because malignant ovarian germ cell tumor commonly affects young women. Although conservation is the standard for early stage, it becomes more debatable as the disease progresses to more advanced stages.

Aim: Report the case of a patient with an International Federation of Gynecology and Obstetrics Stage IIIc malignant ovarian germ cell tumor, who had conservative surgery and chemotherapy with a good fertility outcome.

**Case presentation:**

A 23-year-old North African woman with a left malignant ovarian germ cell tumor stage IIIc was treated by left adnexectomy and omentectomy followed by chemotherapy. A 15-year follow-up showed no signs of relapse, and she completed three full-term natural pregnancies.

**Conclusions:**

Malignant ovarian germ cell tumor is a rare ovarian tumor with a good prognosis. It is usually associated with a good fertility outcome in early stages. However, due to the rarity of the disease in advanced stages, the fertility outcome for this group of patients is not clear. This lack of data surrounding advanced stages points to the need for a meta-analysis of all published cases.

## Background

Malignant ovarian germ cell tumor (MOGCT) is a rare disease. It represents 5 to 7% of all ovarian malignancies [1, 2]. There is a variety of histologic types: dysgerminoma, immature teratoma, yolk sac tumor, choriocarcinoma, polyembryoma, and mixed MOGCT. They differ from epithelial ovarian cancer in two aspects: the younger age of the affected population and their good prognosis. The latter is explained by the higher number of diagnoses at early stages and their high chemosensitivity [3, 4].

Fertility-sparing surgery (FSS) is the cornerstone of treatment in early stage disease. In advanced stage MOGCT, conservative surgery for young patients who desire pregnancy can be justified after discussion with the patient [4].

Patients with MOGCTs have a relative good pregnancy rate even in advanced stages [5].

Our aim is to report a case of advanced MOGCT with a 15-year survival and an excellent fertility outcome of three spontaneous pregnancies.

## Case presentation

A 23-year-old North African woman, with no past personal or familial medical history, requested a consultation for acute abdominal pain and deterioration of her general status.

After a physical examination, she was found to be afebrile with a whole abdominal tenderness but no signs of peritonitis. A vaginal examination found a mobile tender mass in the pouch of Douglas. The remaining part of the physical examination was normal. A pelvic ultrasound showed a 22 × 15 cm simple left ovarian cyst. In view of her acute abdominal pain, she was scheduled for an emergency cystectomy. A 25 cm left ovarian tissular mass with external vegetation associated with a moderate ascites and peritoneal implants were observed; the rest of her abdominal cavity appeared normal.

A left adnexectomy was also conducted. The frozen section showed a malignant germ cell tumor. The surgery was completed by omentectomy and excision of all the peritoneal implants. A final histologic examination showed a mixed MOGCT associating a seminomatous component to a yolk sac tumor. The peritoneal implants were metastatic.

It was at that time that she was referred to our center.

She underwent chemotherapy consisting of bleomycin, etoposide, and cisplatin (BEP), with a complete clinical and radiological response after four courses of BEP. Finally, she underwent six well-tolerated injections of BEP with no chemo-induced amenorrhea.

She was then contacted for follow-ups for the next 16 years. They were initially conducted every 4 months, including physical examinations and tumor markers. Two years post-treatment, follow-ups were conducted every 6 months for the next 3 years. She then received annual follow-ups for the remainder of the time. An abdominopelvic sonography was also conducted annually. The tumor markers were negative during all follow-ups and there have been no signs of relapse to this date.

She also reported three full-term natural pregnancies, occurring respectively 4, 6, and 10 years after the end of the treatment.

## Discussion

Through this case report we have observed that, despite the advanced stage, FSS can offer both oncologic safety and good reproductive outcome.

### Oncologic safety

Because of the rarity of advanced stage MOGCTs, they comprise approximately 20 to 30% of all MOGCTs [6], few cases have been reported in the literature. As such, the safety of FSS for this disease is accepted but not yet fully clinically supported.

Alternatively, many studies [4, 7, 8] targeting all stages of development (I to IV) have shown excellent survival outcomes of FSS. This was the case even for advanced stages, and the reproductive outcomes appeared extremely encouraging. Park *et al*. [4] showed that disease-free survival (DFS) and overall survival (OS) were not compromised after FSS in advanced stage (stage II to IV) MOGCTs.

In the study of Low *et al*. [8], the overall mean follow-up duration was 52.1 months, and there were only one out of 18 fatalities (OS = 94.4%). In Perrin *et al*. [7], the 5-year OS rate was seven out of eight patients (88%).

A study published in 2015 by Yang *et al*. [9] examined 104 patients with histologically confirmed MOGCT that were divided into two groups: a fertility-preserving group of 59 cases and a non-fertility-preserving group of 45 cases. This study showed that the mortality rate of the fertility-preserving group was 15.3% lower than that of the non-fertility-preserving group (31.1%; *p* = 0.054). The tumor-free survival rate in the fertility-preserving surgery group was 79.7%, which was higher than that of the non-fertility-preserving surgery group which was 57.8% (*p* = 0.016). The 5-year progression-free survival (PFS) rates in the fertility-preserving surgery group and the non-fertility-sparing group were of 67.6 and 63.3%, respectively.

Ertas *et al*. [10] showed that in 42 cases of patients with MOGCT treated by FSS the OS rates were 100% for patients with pure dysgerminoma, 81.4% for patients with non-dysgerminoma tumor, 97.4% for early stage disease, and 77% for advanced stage disease, respectively.

Based on these results, FSS is widely accepted as a surgical approach for young women desiring pregnancy.

### Reproductive outcome

In previous studies [4, 11–13], most patients had normal menstruation and their ovarian function was very promising after BEP chemotherapy.

Park *et al*. [4] reported that 15 out of 20 patients (75%) with MOGCT, all stages combined, succeeded in their attempts to conceive: 21 pregnancies were reported with 13 live births. As such, reproductive outcomes are encouraging after FSS, even in patients who received adjuvant BEP chemotherapy.

In another study, Brewer *et al*. [5] showed that 71% of patients maintained their normal menstrual function during and after chemotherapy, including patients with advanced stage disease.

In their study, Yang *et al*. [9] found that in the fertility-preserving group of 39 patients that attempted to conceive, there was a total of 31 successful pregnancies. The pregnancy rate was 79.5%, and the total number of pregnancies was 37.

In another study conducted by Ertas *et al*. [10], 58% of those receiving chemotherapy were amenorrheic during treatment while 85% resumed regular menses after completing chemotherapy. The fertility rates were 77% for those who received adjuvant chemotherapy after FSS and 62.5% for those who did not receive it.

These studies point to the expectation that advanced stage MOGCT can be cured with maintenance of normal reproductive function when treated with conservative surgery and BEP chemotherapy.

## Conclusions

MOGCT is a rare disease, usually affecting young patients. Therefore, the preservation of fertility should be the aim for the gynecologic oncologist. This case, as with the few other cases reported in the literature, showed that conservative treatment in advanced stages can yield good results as it relates to oncologic safety and reproductive outcomes. As such, treatment can be presented as an option to patients after offering a clear explanation and obtaining full consent. On the other hand, and due to the rarity of research on advanced stages of the disease, the need for more case series and meta-analyses is crucial to obtain a higher grade of recommendation to FSS in advanced stage MOGCT.
